# Misinterpretation with norm-based scoring of health status in adults with type 1 diabetes

**DOI:** 10.1186/1477-7525-4-15

**Published:** 2006-03-16

**Authors:** Alison L Supina, David H Feeny, Linda J Carroll, Jeffrey A Johnson

**Affiliations:** 1 Centre for Health and Policy Studies, University of Calgary, Calgary, AB, Canada; 2 Institute of Health Economics, Department of Economics, University of Alberta, Edmonton, AB, Canada; 3 Kaiser Permanente Northwest Center for Health Research, Health Utilities Inc., Dundas, ON, Canada; 4 Department of Public Health Sciences, Faculty of Medicine, University of Alberta, Edmonton, AB, Canada; 5 Institute of Health Economics, #1200 10405 Jasper Ave NW, Edmonton, Alberta, T5J 3N4 Canada

## Abstract

**Background:**

Interpretations of profile and preference based measure scores can differ. Profile measures often use a norm-based scoring algorithm where each scale is scored to have a standardized mean and standard deviation, relative to the general population scores/norms (i.e., norm-based). Preference-based index measures generate an overall scores on the conventional scale in which 0.00 is assigned to dead and 1.00 is assigned to perfect health. Our objective was to investigate the interpretation of norm-based scoring of generic health status measures in a population of adults with type 1 diabetes by comparing norm-based health status scores and preference-based health-related quality of life (HRQL) scores.

**Methods:**

Data were collected through self-complete questionnaires sent to patients with type 1 diabetes. The RAND-36 and the Health Utilities Index Mark 3 (HUI3) were included.

**Results:**

A total of 216 (61%) questionnaires were returned. The respondent sample was predominantly female (58.8%); had a mean (SD) age of 37.1 (14.3) years and a mean duration of diabetes of 20.9 (12.4) years. Mean (SD) health status scores were: RAND-36 PHC 47.9 (9.4), RAND-36 MHC 47.2 (11.8), and HUI3 0.78 (0.23). Histograms of these scores show substantial left skew. HUI3 scores were similar to those previously reported for diabetes in the general Canadian population. Physical and mental health summary scores of the RAND-36 suggest that this population is as healthy as the general adult population.

**Conclusion:**

In this sample, a preference-based measure indicated poorer health, consistent with clinical evidence, whereas a norm-based measure indicated health similar to the average for the general population. Norm-based scoring measure may provide misleading interpretations in populations when health status is not normally distributed.

## Background

Interpretation of health-related quality of life (HRQL) instrument scores and differences between subgroups is critical in the wide application of such tools [1,2]. Interpretation can, however, be hampered due to various interpretation methods/criteria, differences between measure development and scoring, and differing perspectives (individual versus population) [2,3] HRQL scores can be interpreted statistically or clinically. While statistical interpretation is rather straightforward, clinical interpretation can be more problematic as *a priori *criteria for these interpretations may be vague at best, if present at all. Various operational definitions of scoring and interpretation (e.g., norm or distribution-based versus anchor-based) can lead to difficulties when comparing HRQL scores results between studies and between groups versus individuals [3,4]. Exploration of norm-based versus anchor-based interpretation of HRQL differences can help to illuminate the strengths and limitations of the measures used.

Generic HRQL measures are intended for general use, irrespective of disease state, population or treatment [5]. These measures can also be used in healthy people in the general population and in patient populations. Appropriate use of generic measures in disease specific populations depends on whether the instrument covers the relevant domains, with an appropriate domain continuum, for the population's disease. Generic measures of HRQL have an advantage over disease-specific measures in that they permit comparisons of the impact of various diseases on multiple dimensions of HRQL and allow comparisons across conditions or populations. Specific measures have the advantage of focusing on issues of particular concern to patients with the disease [6]. Also, they may be better able to identify functional impairments arising for the illness under study and may be more sensitive to small changes in health resulting from treatment than generic HRQL measures [7]. For these reasons, patients and clinicians often tend to prefer specific measures, as items seem clinically sensible. Disadvantages of disease specific measures are that they may not permit broad comparisons between disease states and they may miss the effects of co-morbidities or treatment side effects. For these reasons, disease specific measures are less informative for resource allocation decision makers and third party payers. Although generic HRQL measures may be less sensitive to disease-specific HRQL burden, they may be expected to distinguish between varying degrees of severity within a condition. Generic measures can be classified into health status profiles and preference-based measures [5].

Profile measures typically reflect an individual's current health status on multiple dimensions or domains and assign a score to each dimension, but do not necessarily provide an overall score to reflect overall HRQL. Profile measures are often derived from psychometric or clinimetric approaches and include key generic health concepts and capture morbidity associated with various health states. However, the scales are not anchored at dead, and therefore they do not include mortality.

Multi-attribute ('indirect') preference-based measures also measure an individual's current health status; however, they then apply a community-derived utility score to value that health state. Preference-based measures offer advantages over profile measures. First, preference measures include the state of "dead", anchored at a value of 0.0, thus integrating both morbidity and mortality. In addition, some preference-based measures allow for negative utility values that reflect health states worse than dead. Preference-based measures also allow an overall score to be obtained, which allows for comparison among diseases and groups as well as an assessment of the overall net effects of disease and intervention. Interpretation of profile and preference based measure scores can differ.

The interpretation of preference-based scores, such as the Health Utilities Index Mark 3 (HUI3) is based on the anchors of "dead" and "full health" and also involves comparison of overall scores with existing external population norms [8]. Profile measures, such as the SF-36 [9] or RAND-36 [10], may utilize a norm-based scoring algorithm where scales have a standardized mean and standard deviation, relative to some reference population (i.e., norms). Although an overall score is not generated in a norm-based scoring system, profile measures and norm-based scoring allow for possible detection of different effects on different dimensions of HRQL. Norm-based scoring is also intended to aid in the interpretation of health status of a sample by having a "built-in" reference (i.e., the 'norm' scores for the population) when applied in any patient population.

Type 1 diabetes is a chronic disease that develops early in adolescence. It can result in acute and long-term complications. Long term microvascular and macrovascular complications account for the majority of the morbidity and mortality associated with diabetes. For these reasons, many middle-aged individuals are heavily burdened with long-term complications and their associated treatments. There is extensive literature based on generic health status/HRQL measurement in diabetes. Previous research with profile and preference-based measures in type 1 and type 2 diabetes have found similar trends in determinants of HRQL burden such as type of treatment and the presence of diabetic complications [11-16]. Despite previous research reporting similar trends between profile and preference-based measures in diabetes, there has been little research comparing the performance and interpretation of these measures in type 1 diabetes.

The objective of this study was to compare the interpretation of norm-based scoring of generic health status and preference-based HRQL measures in an adult type 1 diabetes population.

## Methods

### Study design and sample

This study used a cross-sectional design, with all data collected through self-complete questionnaires mailed to adult type 1 diabetes patients. A second questionnaire was sent to non-responders. Included subjects were adults with clinically diagnosed type 1 diabetes. Subjects had to be eighteen years old at the time of survey completion, be English-speaking, and have a fixed address. All subjects were type 1 diabetes patients being seen at diabetes clinics in Edmonton and Calgary, AB, Canada. Participating endocrinologists and clinic staff provided names and addresses of potential subjects. These patient names and addresses were not pre-screened for any reason by clinic staff. Ethical approval for this study was obtained through the University of Alberta Health Research Ethics Board and the University of Calgary Research Ethics Board.

### Measures

#### Clinical and demographic questionnaire

Subjects completed a sociodemographic and clinical self- report questionnaire. The sociodemographic component of the questionnaire contained questions about their age, sex, marital and occupational status, highest level of education, and main activity in the last twelve months. The clinical self-report component of the questionnaire contained questions regarding diagnosis, duration, glycemic control and advancement of diabetes. Also, it contained questions regarding signs and symptoms of diabetic complications and a self-report of common co-morbidities, adopted from the National Population Health Survey (Statistics Canada) [17].

#### Health Utilities Index Mark 3 (HUI3)

The HUI3 is preference-based multi-attribute utility measures of HRQL, which assess multiple domains of health status, and assigns a valuation to each health state, based on community preferences for health states [8]. Health states are classified by a set of dimension or attributes of HRQL, with a number of different levels for each attribute. HRQL is classified by eight attributes: vision, hearing, speech, ambulation, dexterity, emotion, cognition, and pain. In the HUI3 system, each of the eight attributes has five or six different levels; these levels describe 972,000 unique HUI3 health states [8]. Overall utility scores on the HUI3 range from -0.36 to 1.0, where -0.36 represents the worst possible HUI3 health state, 0.0 represents dead, and 1.0 represents full health [8].

Differences greater than 0.03 on the HUI3 overall scores are considered to be clinically important [18,19]. In a population health survey, overall HUI3 scores were found to have a test-retest reliability using an intra-class correlation coefficient (ICC) of 0.77 in one-month follow-up [20]. Other studies of disease specific patient populations such as multiple sclerosis, hip fracture and rheumatoid arthritis have reported HUI3 scores to have test-retest reliability using ICCs ranging from 0.72 to 0.87 [20-24].

The HUI3 may be useful in studying HRQL in diabetes because of several attributes that would likely be affected by the severity of diabetes and diabetic complications [19,25]. Specifically, diabetic complications such as amputation and peripheral neuropathy may affect the ambulation and dexterity attributes of the HUI3. In addition, neuropathy and myopathy may affect the pain and discomfort and dexterity attributes of the HUI3. Retinopathy may affect the vision attribute and nephropathy may affect the ambulation and pain attributes of the HUI3. While the measurement properties of the HUI3 have been explored in type 2 diabetes [19,25], no experience existed with regard to type 1 diabetes.

In addition to containing attributes relevant to diabetes, the HUI3 has relevance as a reference standard for the general Canadian population, as the HUI3 has been included in all recent national health surveys. Recent experience with the HUI3 in the general population (from 1996–1997 National Population Health Survey (Cycle 2) [26] provided an overall adjusted HUI3 score of 0.88 (95%CI: 0.87–0.89) for respondents with type 2 diabetes alone (adjusted for age, sex, education and number of medical conditions) [27]. This was statistically significantly lower than the score of 0.92 (95%CI: 0.92–0.92) (p < 0.001) for subjects without diabetes; the difference is also clinically important [25].

#### RAND-36 health status inventory

The RAND-36 is a commonly used health profile instrument [8]. It was designed to evaluate 8 areas of behavior or experience including physical functioning, role limitations due to physical problems, bodily pain, general health perceptions, vitality, social functioning, and role limitations due to emotional problems, mental health and health transition [8]. In addition, two summary scores representing physical (Physical Health Composite – PHC) and mental (Mental Health Composite- MHC) health are generated [8]. Although the RAND-36 employs the same items as the SF-36, the methodology used to derive the composite scores for the RAND-36 differs from the SF-36. Specifically, the RAND-36 uses an oblique rotation, rather than the orthogonal rotation employed in the SF-36. The orthogonal rotation used for SF-36 is designed to result in independent uncorrelated composite scores [10]. The oblique rotation used for the RAND-36 allows the two summary scores to be correlated [10]. Also, the domain scores used for composite score construction of the RAND-36 are only those associated with either physical or mental health. In contrast, the SF-36 uses all domain scores in the construction of both the physical and mental composite scores. In the SF-36, mental domains have a negative effect and physical domains have a positive effect on the physical composite scores and vice versa for the mental composite score.

For these reasons, it is felt that the RAND-36 provides a more rational and clinically sound scoring system for HRQL. Recent evidence suggests that the different scoring approaches will affect the validity of the summary scores, as represented by the RAND-12 and SF-12 [29,30].

The RAND-36 (or the related SF-36) has been frequently applied in the assessment of health status in diabetes [20,22-24]. The RAND-36 summary scores are T-score norm-based scoring approaches; therefore, interpretation of these T-scores is based on a general US population mean of 50.0, with a standard deviation of 10.0 [8]. It is suggested that a minimum difference of three to five points on any given scale may be considered clinically important [31].

It is important to note that there is substantial overlap in the domains of health status covered by HUI3 and the RAND-36. For instance, both measures include physical functioning, bodily pain, and mental health. Of course there are also domains covered by one measure but not the other such as vitality (RAND-36) and vision, hearing, and speech (HUI3).

### Data analysis

HRQL measures were scored according to the developers' guidelines. Descriptive statistics were calculated to present the minimum, maximum, median and mean (SD) for the HUI3 and RAND 36 scores in this sample. The respondent sample was described by self-reported demographic and clinical characteristics. We compared descriptives and distributions for the HUI3 and RAND-36. Overall measure scores were also compared using Pearson's correlations. Histograms were generated for comparisons of score distributions.

## Results

A total of 216 questionnaires were returned, for an overall response rate of 61.0%. Of the 216 respondents who met all study inclusion criteria, the majority were female (127, 58.8%) and were married or in a partnership (131, 60.6%) (Table 1). The highest level of completed education for most respondents included high school (19.4%), some college education (19.9%), and a college degree (19.0%). Working (full or part time employment) was the main activity in the last twelve months for the majority of respondents (58.3%). Total household income last year for the sample ranged from ≤ $10 000 (9.7%) to ≥ $70 000 (30.6%).

**Table 1:** Sample demographic characteristics.

**Characteristic**	**n**	Total*
**Age (yrs) – mean (SD)**	215	37.13 (14.28)
**Sex**	216	
Female		127 (58.8)
**Marital Status**	216	
Single		69 (31.9)
Married/In a partnership		131 (60.6)
Separated/Divorced		13 (6.0)
Widowed		3 (1.4)
**Highest Level of Completed Education**	216	
Less than high school		16 (7.4)
High school		42 (19.4)
Some college		43 (19.9)
College degree		41 (19.0)
Some university		27 (12.5)
University degree		40 (8.5)
Other		7 (3.2)
**Main Activity in Last 12 months**	216	
Working		126 (58.3)
Looking for work		11 (5.1)
Keeping house		18 (8.3)
Student		30(13.9)
Disability		16 (7.4)
Retired		15 (6.9)
**Total Household Income Last Year**	196	
≤ $10 000		19 (9.7)
$10 000 – 29 999		44 (22.4)
$30 000 – 49 999		37 (18.9)
$50 000 – 69 999		36 (18.4)
≥ $70 000		60 (30.6)

*n (%) unless otherwise specified

Respondents had a mean age of 37.1 (SD 14.3) years, mean (SD) duration of diabetes of 20.9 (SD 12.4) years (median of 19.0 years), with a median age of diagnosis of 12.0 years (Table 2) The majority of respondents were at a normal weight (47.9%) at diagnosis, with 92.9% of individuals starting insulin therapy within 3 months of diagnosis and a median of 4 insulin injections per day. These clinical characteristics affirm that the subjects in this sample would be considered to have type 1 diabetes.

**Table 2:** Sample clinical characteristics.

**Characteristic**	**n**	Total*
**Duration of Diabetes (yrs) – mean (SD)**	215	20.91 (12.43)
**Age at Diagnosis (yrs) – median (SD)**	215	12.0
**Weight at Diagnosis**	211	
Underweight		89 (42.2)
Normal weight		101 (47.9)
Overweight		21 (10.0)
**Started insulin within 3 months**	210	195 (92.9)
**Insulin injections per day -median (min, max)**	214	4.0 (1.0–5.0)
**Presence of Diabetic Complications**		
Retinopathy	215	88 (40.7)
Neuropathy/Peripheral vascular disease	213	73 (33.8)
Cardiovascular disease	215	55 (22.5)
Nephropathy	214	40 (18.5)
**Frequency of Diabetic Complications**		
No Diabetic complications reported	216	82 (38.0)
1 Diabetic complication reported	216	56 (25.9)
2 Diabetic complications reported	216	44 (20.4)
≥ 3 Diabetic complications reported	216	34 (15.7)
**Number of Co-morbidities Reported**^†^	216	
No Co-morbidities Reported		118 (54.6)
1 Co-morbidity Reported		53 (24.5)
2 Co-morbidities Reported		25 (11.6)
≥ 3 Co-morbidities Reported		20 (9.3)
**Most prevalent co-morbidities (median)**	166	1.0
Thyroid condition	167	35 (21.2)
Arthritis/rheumatism	167	28 (16.8)
Asthma	166	19 (11.4)

*n (%) unless otherwise specified

^† ^Medical conditions considered to be complications were not included as a co-morbidity

The self-reported presence of diabetic complications is shown in Table 2. Based on the *a priori *study criteria for the presence of diabetic complications, the prevalence of diabetic complications in this sample was: retinopathy/diabetic eye disease (40.7%); neuropathy/peripheral vascular disease (33.8%); cardiovascular disease (25.5%); nephropathy (8.5%); the majority of the sample (62.0%) reported one or more diabetic complication(s). Thyroid condition, arthritis/rheumatism, and asthma were the most prevalent co-morbidities reported.

**Figure 1:**
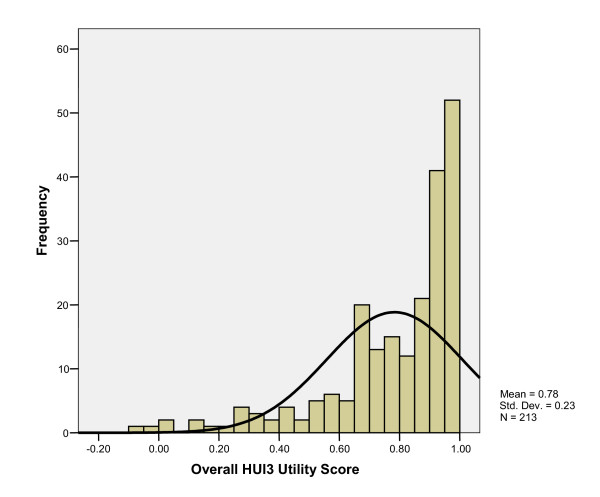
Histogram of Overall HUI3 Scores.

**Figure 2:**
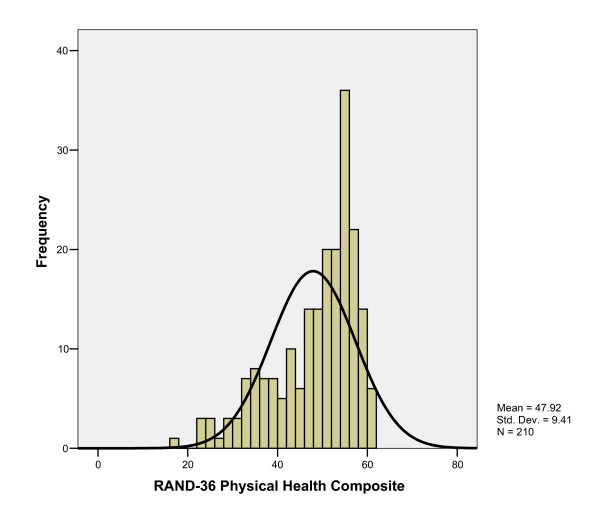
Histogram of RAND-36 Physical Health Composite Score.

**Figure 3:**
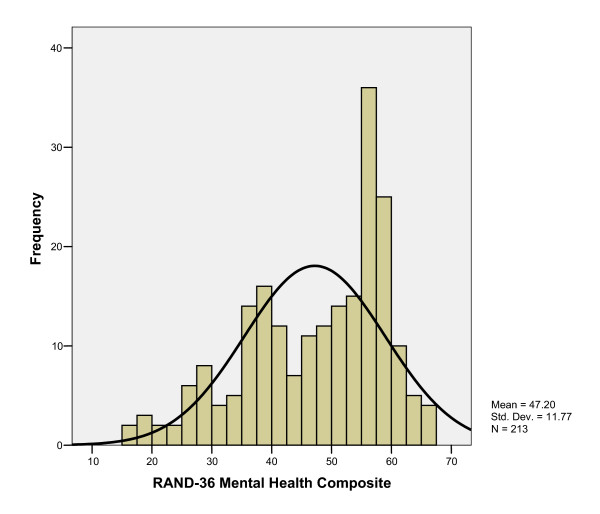
Histogram of RAND-36 Mental Health Composite Score.

Respondent's overall mean (± SD) HUI3 score was 0.78 ± 0.23 (Table 3). RAND PHC and MHC composite scores were 47.92 (± 9.41) and 47.20 (± 11.77), respectively (Table 3). Overall HUI3 measure scores were strongly correlated with RAND-36 PHC and MHC scores (r = 0.68 and 0.71, respectively). Histograms of overall health status scores show the distribution of scores to be not normally distributed, with substantial skew to the left for both measures (Figures 1, 2, 3). The distributions of the RAND-36 summary scores, particularly the MHC, approach normality more than the distribution of HUI3 scores; however, all distributions remained skewed.

**Table 3:** Descriptive statistics for HRQL measure overall scores.

**Score**	**N**	**Mean**	**SD**	Min	**Max**	**Median**	**IQR**
HUI3 Overall	213	0.78	0.23	-0.08	1.00	0.85	0.68–0.95
RAND-36 PHC	210	47.92	9.41	16	61	51.00	39–63
RAND-36 MHC	213	47.20	11.77	15	66	50.00	31–69

In this sample, mean HUI3 and RAND-36 scores reflect a HRQL burden similar to that previously reported for type 2 diabetes [20,21]. In addition, HUI3 scores in this sample reflect a large HRQL burden, in comparison to a previously reported general Canadian population (age and sex adjusted) norm of 0.90 [26]. Interestingly, the RAND-36, a norm-based scoring health status measure, did not reflect a similar HRQL burden in this sample. Norm-based interpretation of RAND-36 PHC and MHC scores suggest that this population is as healthy as the average general Canadian population. Although the RAND-36 summary scores do identify a proportion of individuals reporting substantial burden, the mean scores are high enough to be interpreted within the normal range for the general population.

## Discussion

Distribution-based interpretation of RAND-36 scores is challenging in this study. RAND-36 PHC and MHC scores of 47.9 and 47.2, respectively, suggest that the sample of type 1 diabetic subjects is approximately as healthy as the general US population. We find this interpretation troublesome, as our anchor-based interpretation of HUI scores show HRQL in adults with type 1 diabetes to be lower than that of the general Canadian population. It would seem logical to accept this second interpretation, given the prevalence of diabetic complications and co-morbidities in this sample.

Further analysis of the distribution of HUI and RAND-36 scores demonstrate that, in fact, scores for all measures were not normally distributed, with substantial skew to the left; a distributional-based approach assumes scores to be normally distributed. Here, distributional-based interpretation of RAND-36 scores may lead to misinterpretation of the HRQL burden associated with type 1 diabetes, as clinical evidence and other HRQL measures would suggest HRQL is lower than in the general population. When considered relative to the HUI3 in this study, because of the strong correlations between overall summary scores, it appears that the RAND-36 summary scores have distorted the interpretation of the HRQL burden by imposing a normal distribution on non-normally distributed data.

Alternative explanations for differences between overall measure scores need to be considered. The differences in HRQL burden may be a result of differences in item content between measures. HUI3 may be more sensitive to diabetic complications, such as the most prevalent complication of retinopathy. This may increase the HRQL burden as measured by the HUI3 relative to the burden as measured by the RAND-36. However, with respect to the HUI3 single-attribute utility score (SAUS) for vision, 95.8% of the sample reported a vision SAUS of ≥ 0.95. Thus it is unlikely that differences in item content explain the differences in mean scores between the measures. Distribution of other SAUS for the HUI3 (i.e., hearing, speech, ambulation, dexterity) were similar to those of the vision SAUS. It should be noted that the differences between PHC and MHC scores for the sample and population norms approach a clinically important difference of 3. However, the difference between sample mean and population norms for HUI3 (diff = 0.14) is nearly 5 times the clinically important difference for the HUI3 overall score [8,31].

These results call into question the usefulness of norm-based scoring in situations where the health of a population is unlikely to be normally distributed. This may be problematic in clinical situations, where prognostic and therapeutic decisions are guided by interpretation of the HRQL burden revealed by the HRQL measure, often based on the mean scores of HRQL measures. Misinterpretation of norm-based scores leading to possible underestimation of HRQL burden, as seen in this analysis, may inappropriately inform health research allocation and policy makers. For this reason, it is important that additional descriptive statistics (e.g., median, standard deviations, quartiles cut points) should be displayed when interpreting HRQL scores.

We recognize several limitations in this study. First, all data and comparisons were cross-sectional. Longitudinal assessments would provide more valid and reliable information regarding the long-term HRQL of this population. It should be recognized that all clinical data were based on patient self-report. However, it should be expected that respondents were motivated to provide valid answers on information about aspects of their lives, which are of high personal relevance to them [26]. Previous studies have shown good agreement between administrative claims, medical records or physician report and self-report for chronic conditions, particularly for those conditions with clear diagnostic criteria, such as diabetes, thus allowing for useful estimates of population prevalence for these conditions [32-36] Also, all self-report co-morbidities were based on a dichotomous response of yes/no therefore; we were not able to capture the severity of reported co-morbidities and complications. Previous research with generic preference-based measures in diabetes shows the presence of diabetic complications (particularly microvascular complications), the intensity of diabetes treatment, and the presence of co-morbidities result in larger HRQL burdens [9,11-15,37].

Lastly, as with all mail-out self-report questionnaires, the issue of responder bias is an important consideration. It is unknown if non-responders were significantly different from responders; therefore, measurement of responder bias in this study was not possible. Given the distribution of sample demographics and clinical characteristics (i.e., prevalence of complications and co-morbidities, insulin use, age and weight at diabetes diagnosis) we feel that this sample can be considered representative of a mainly urban-dwelling population of adults with type 1 diabetes, when compared to Alberta census reports for Edmonton and Calgary (2001), where the majority of the population ranges in age from 25–54 years, have a trade or non-university certificate/diploma (31.2% and 30.1%, respectively) with a household income of $60,000 and over (41.9% and 48.8%, respectively) [38]. Also, the prevalence of diabetic complications in our sample is similar to those previously reported for individuals with a duration of diabetes of twenty-five years or greater where, the prevalence of complications are estimated at 10–30% for cardiovascular and/or peripheral vascular disease, 25–45% for nephropathy, 50% for neuropathy, and 50–70% for some degree of retinopathy [39-43].

## Conclusion

In this sample, a preference-based measure indicated poorer health, consistent with clinical evidence, whereas a norm-based measure indicated health status similar to that of the general population, despite evidence to the contrary. Norm-based scoring may lead to misinterpretation of HRQL norm-based scores.

## Competing interests

It should be noted that David Feeny has a proprietary interest in Health Utilities Incorporated, Dundas, Ontario, Canada. HUInc. distributes copyrighted Health Utilities Index (HUI) materials and provides methodological advice on the use of HUI.

## Authors' contributions

AS was involved in all aspects of this study particularly study design, data collection, data analysis, data interpretation, presentation and manuscript preparation. JJ, DF, and LC provided guidance and support in all areas of this project, particularly in study design, data interpretation and manuscript preparation. This study was conducted as a thesis project for AS, under the supervision of JJ. All authors read and approved the final manuscript.
